# Forensic Speaker Verification Using Ordinary Least Squares

**DOI:** 10.3390/s19204385

**Published:** 2019-10-10

**Authors:** Thyago J. Machado, Jozue Vieira Filho, Mario A. de Oliveira

**Affiliations:** 1 Campus of Ilha Solteira, São Paulo State University (UNESP), São Paulo 15385-000, Brazil; 2 Telecommunications and Aeronautical Engineering, São Paulo State University (UNESP), São João da Boa, Vista SP 13876-750, Brazil; jozue.vieira@unesp.br; 3 Automation and Control Engineering, Mato Grosso Federal Institute of Technology, Cuiabá 78005-200, Brazil; mario.oliveira@cba.ifmt.edu.br

**Keywords:** forensic speaker comparison, forensic phonetics, voice processing, ordinary least squares (OLS), linear predictive coding (LPC)

## Abstract

In Brazil, the recognition of speakers for forensic purposes still relies on a subjectivity-based decision-making process through a results analysis of untrustworthy techniques. Owing to the lack of a voice database, speaker verification is currently applied to samples specifically collected for confrontation. However, speaker comparative analysis via contested discourse requires the collection of an excessive amount of voice samples for a series of individuals. Further, the recognition system must inform who is the most compatible with the contested voice from pre-selected individuals. Accordingly, this paper proposes using a combination of linear predictive coding (LPC) and ordinary least squares (OLS) as a speaker verification tool for forensic analysis. The proposed recognition technique establishes confidence and similarity upon which to base forensic reports, indicating verification of the speaker of the contested discourse. Therefore, in this paper, an accurate, quick, alternative method to help verify the speaker is contributed. After running seven different tests, this study preliminarily achieved a hit rate of 100% considering a limited dataset (Brazilian Portuguese). Furthermore, the developed method extracts a larger number of formants, which are indispensable for statistical comparisons via OLS. The proposed framework is robust at certain levels of noise, for sentences with the suppression of word changes, and with different quality or even meaningful audio time differences.

## 1. Introduction

Voice recognition is a process to identify the interlocutor and/or discourse performed. This is derived from specific information extracted from speech, and can be conducted either manually or automatically. Manual recognition is subjective, intuitive, and subject to hearing and recognition of human patterns. The automatization of this process aims to bring objectivity and speed to recognition. Using information speech waveforms, it is possible to design a tool capable of recognising what was said in the discourse, or to identify the interlocutor. The applications are diverse, and mainly linked to the control of access to services by voice, including the following: phone activation by voice; banking services through a phone network; phone shopping; database access services; information and booking services; voicemail; classified information security control; and remote access to computers [1]. Voice recognition can be applied in two ways: discourse recognition (identifying speech or phonetics of the signal), and speaker recognition (to identify the speaker) [2]. Despite presenting different goals, both discourse and speaker recognition deal with the same dilemmas. Accent, noise, and phonetics are some of the factors that make pattern recognition tools applied to voices a difficult task [3].

Within the area of speaker recognition, it is usual to divide the subject into identification and verification [4]. Speaker verification occurs when the system must compare a confronted discourse with a base (reference) discourse in such a way that verifies whether both have been produced by the same speaker. Regarding identification, this compares the confronted discourse with a database and/or voices from a series of individuals. Most of the applications in which voice is used to confirm identity are classified as speaker verification [5]. Thus, the recognition system must identify who is the most compatible with the confronted voice from the pre-selected individuals [6]. It should be noted that speaker identity correlates to the physiological and behavioural characteristics of the individual speech production system. This may link such characteristics to the cepstral coefficients and voice regression [7].

It relates one application for speaker recognition to forensic phonetics. Forensic voice comparison is based on comparing a recording of an unknown criminal’s voice (the questioned piece or trace) and a recording of a known suspect’s voice (the comparison piece) [8]. For forensics, the decision-making of speaker verification must present a high level of confidence. From a critical review of the research literature, several related papers were found. These found methods usually take advantage of formants as a powerful speaker verification tool. For example, in [9] the authors used audio excerpts of 3–5 s, then extracted 5 formants and compared them to the spectral fragments to identify differences and coincidences. When compared to the same excerpt, a 1% false positive was obtained, which increased to 2% at the moment they were compared to different excerpts. This method did not use any statistical techniques in the model; only visual techniques were used with an error ratio that was low. Another study also used formants to identify the speaker [10]. They applied a technique based on spectral moments, and hit a 10% to 20% error rate on speaker verification. Speaker recognition using formants was also employed in [11], by semi-automatically extracting only the F1, F2, and F3 formants. The authors used the Gaussian mixture model (GMM), which (in the best-case scenario) achieved an error rate of 3% when using 3 formants and their corresponding bandwidths. The work in [12] also presented a statistical approach for speaker verification in the field of forensic phonetics. They extracted the formants (F0, F1, F2, F3 and F4) by using Praat software, and posteriorly applied the Mahalanobis distance together with statistical distribution to identify the formants that best match the voice signature. As a result, they achieved the smallest error rate close to 4%. Likewise, another approach used Praat software and was based on the likelihood rate and coincidentally, they hit an error rate of 4% [13]. A semiautomatic speaker verification system based on a comparison of formants values, statistics of phone call time-spans, and melodic characteristics, was proposed in [14]. The accuracy of the recognition system was 98.59% on a database containing recordings of males, and 96.17% on a database containing recordings of females. They used a database with recordings lasting from 3 to 5 min.

Neural networks (NNs) have played an important role in speaker recognition. Recently, some techniques for speaker verification have used deep neural networks (DNNs). In [15], a revision containing nine different techniques by employing DNN was presented. The results hit 0.2% and 0.88% for comparisons based on a dependent and independent text, respectively. Although these were good results, they did not take into account the time processing cost, as the method needs hours of learning. It is necessary for the announcer to record the maximum possible amount of different vocabulary in a row, with the aim of allowing the NN to achieve an enhanced success rate. The work in [16] demonstrated a method based on a combination of NN and fuzzy logic to achieve speaker recognition. Whilst positive results were obtained (up to 78%), the computational cost in training was high. In [17], an applied comparison of voice recognition on a large scale was conducted [17]. The database contained over 1 million statements from over 6000 speakers, and they achieved an error rate of 3.95% using DNN [18]. The presented results are excellent if considered as a filtering technique for several speakers. However, it could not be considered for the forensic field where precision is paramount. Similarly, [19] presented a recognising speaker approach, by using a combination of the Gabor filter, statistics, and convolution neural networks (CNNs). They achieved an error rate of only 0.58% at a processing speed of 0.3456 seconds. Despite these good results, none of the methods presented here were capable of a 100% hit rate.

It can be observed in the papers reviewed that only tests with independent and dependent texts were performed. They did not use scenarios such as temporisation of uneven speech, different sound quality, and noise insertion, which could affect the accuracy of the methods. According to [8], the question of reliability remains a major challenge, particularly for forensic voice comparison systems where numerous variation factors like duration, noise, linguistic content, or within-speaker variability are not taken into account. Furthermore, speaker recognition systems exhibit a decrease in performance when the user is under emotional or stress conditions [20]. In [21], the author indicated that the accuracy is subject to disruption by background noise and differences in speaking style, both of which may play a role in cases involving voice discrimination. In this sense, [22] proposed to identify the speaker considering the influence of noise. They used a mixed method based on the multi-taper gammatone Hilbert envelope coefficients (MGHECs) and multi-taper chirp group delay zeros-phase Hilbert envelope coefficients (MCGDZPHECs). They claimed a significant improvement in performance under noisy conditions, when compared to conventional mean Hilbert envelope coefficients (MHECs). According to the results obtained in [18], hybrid models in speaker comparison enhance the chances of success. Hence, they took advantage of the pipelined near real-time speaker recognition architecture that enhances the performance of speaker recognition. This is achieved by exploiting the advantages of hybrid feature extraction techniques containing the features of Gabor filters, CNNs, and statistical parameters as a single matrix set. Based on this, the method developed herein performs Hanning windowing, the Fourier transform, linear predictive coding (LPC), and ordinary least squares (OLS). 

Therefore, this paper proposes the use of OLS as a speaker recognition method for forensics. The focus here is speaker verification from the samples collected on confrontation (bearing in mind that there is an absence of a voice database in Brazil, considering the Brazilian authorities such as the Federal Police and the Criminal Institutes of the Brazilian States). Speaker recognition in Brazil still presents decision-making based on the subjective analysis of results using unreliable techniques [6]. Therefore, the technique proposed in this paper establishes a different method for recognising speakers (taking account of those found in the literature) that can be applied to the Brazilian Portuguese speaker. First, this study submits the discourses to formants and pitch extraction using linear predictive coding (LPC). After extraction of these speech characteristics, the technique analyses the contested audio, and performs a confrontation with pre-selected patterns, using OLS [19]. As a tool of speaker verification, OLS is likely to find a profile for the contested audio and later to establish similarity between the profile and the speech patterns of other individuals. This way, by using the similarity grade of the parameters extracted from the speeches, it can be possible to determine which individual has a more likely chance of being recognised as the speaker of the confronted speech. After the performed tests, attempts were also made to analyse the robustness of the technique regarding textual discourse. In short, the main contributions of this paper are as follows:

- This study developed a novel method suitable for speaker verification, which is an unpublished method that takes advantage of the combination of the formants, LPC, and OLS to generate results for decision-making in a forensic context;
- The robustness of the developed model is demonstrated by generating positive results, even with atypical situations such as noise, uneven speech time, quality, and textual independence;
- All scenarios that were preliminarily tested have indicated a 100% success rate, considering a limited dataset (Brazilian Portuguese), reducing the possibility of false positives.

The remainder of this paper is organised as follows. First, the main theoretical foundations of the work are addressed. Second, the developed method is presented, highlighting the combination of the LPC and OLS. Third, the results are presented, followed by a comparison with other approaches, and finally, the paper concludes by highlighting remarks on the developed approach.

## 2. Signal Analysis

### 2.1. Fourier Transform and Windowing

The Fourier transform is a method for representing a sign by its frequency components [23]. This comes from the representation of the Fourier series, which shows it is possible to represent any signal by the sum of simple waves, such as sines and cosines [24]. With regard to discrete-time processing, the discrete Fourier transform (DFT), containing N-points of any signal x[n] is defined as follows [25]:

(1) $$X\left[k\right]=\sum_{n=0}^{N-1}x\left[n\right]\;e^{\frac{-j2\pi kn}{N}},$$

where *k* = 0, …, *N* − 1. Through this operation, it is possible to represent the values in x through the magnitude of the components of this signal in each frequency value. This is a valuable tool in a data processing context, once this makes clear the characteristics of the signal that are not possible to observe in a time domain. The analysis of frequency characteristics is known as spectral analysis [24]. For audio analysis, spectral analysis is a valuable tool, because it extracts information about the origin and properties of the audio file, assessing the most prominent frequency components of the signal. These most prominent frequencies are known as formants and they work as a species of a particular signature for each presenter, providing the intrinsic properties of the vocal tract for each person [26].

A windowing function comprehends a mathematical operation, and is often used in discrete signal processing [27]. It is used to select a segment of the signal according to its properties. Thus, windowing functions are widely used, for example, in digital filters, once they can restrict a sign at both time and frequency domains [27]. Furthermore, this provides a smoothening mechanism of interference on the data by reducing distortions caused by the edge effects during spectral decomposition [28]. Accordingly, a series of windowing functions have been used, including: rectangular, triangular, B-sline, Parzen, Welch, Blackman, and Hann [29].

The Hanning windowing function is widely used, particularly because of its effect on smoothening the edge, and because it is commonly applied in audio signal processing [30]. Its equation is given by:

(2) $$w\left(n\right)=0.5*\left[1-\cos\left(\frac{2\pi n}{N}\right)\right]=\sin^{2}\left(\frac{\pi n}{N}\right),$$

where, *w*(*n*) is the Hanning windowing function, *N* is the length of the window, and n is the value throughout the interval 0 ≤ *n* ≤ *N*. If the waveform contains more than one signal with a small difference in frequency, the spectral resolution is important. Here, it is better to choose a window with a narrow main lobe, such as the Hanning window.

### 2.2. Linear Predictive Coding 

LPC is a data compression technique widely used in audio analysis [31] and is an analogue signal codification that comprises the construction of a model for a signal from a linear function of its previous values. It was first introduced in 1984 as a tool for data compression [32]. Using LPC, it is possible to build an envelope that represents the power spectrum of reliable audio signal frequencies simultaneously containing a low bit rate, preserving its characteristics of interest. LPC coefficients are estimated as follows [33]:

(3) $$s_{p}\left(n\right)=\sum_{k}^{K}a_{k}*s\left(n-k\right),$$

where, *a_k_* is the estimated coefficients for the linear model, *n* represents each value of the modulated signal, *s_p_*(*n*) is the predicted sample for a given iteration of the model, *k* is the coefficient, and *K* are the maximum numbers of the coefficients on the model.

The model to be created takes as an input the number of coefficients expected to represent the signal of interest, given by:

(4) $$K=4+\left(\frac{F_{s}}{1000}\right),$$

where *K* is the number of coefficients, and *F_s_* is the sampling frequency. Although the original *s*(*n*) and estimated *S_p_*(*n*) are close, the error *err*(*n*) is given by:

(5) $$err\left(n\right)=s\left(n\right)-s_{p}\left(n\right).$$

To obtain an improved model, the error sum of squares should be minimised. Conversely, taking a high number of coefficients maximises the accuracy of the envelope calculated via LPC, resulting in a more reliable representation of the frequencies’ power spectrum [34]. To get to the roots of the coefficients, the following equation can be used:

(6) $$rts=\sum_{n=1}^{N}s_{p}\left(n\right)*x^{N-n},$$

where, *s_p_* are the coefficients of the polynomial, rts is its root, *n* is the coefficient, and *N* the maximum number of the polynomial’s coefficients.

In this work, the LPC technique is used to determine the formants’ frequencies. Formants are voice energy peaks that set a speaker’s sound profile [34]. Pitches are vibrations of the vocal cords and its modes. It can also be defined as a voice’s waveform, widely publicised as the first formant, which carries the most important information for speaker differentiation. Formants are frequencies that present a more prominent character when the power spectrum of an audio signal’s frequencies are analysed [33]. They are characterised by peaks that appear throughout the spectrum. A number of studies have considered the major contribution of up to five of those frequencies on voice composition, the first being of greater intensity and denominated pitch (or *F0*), and the others (*F1* to *F4*) accordingly [26].

**Figure 1:**
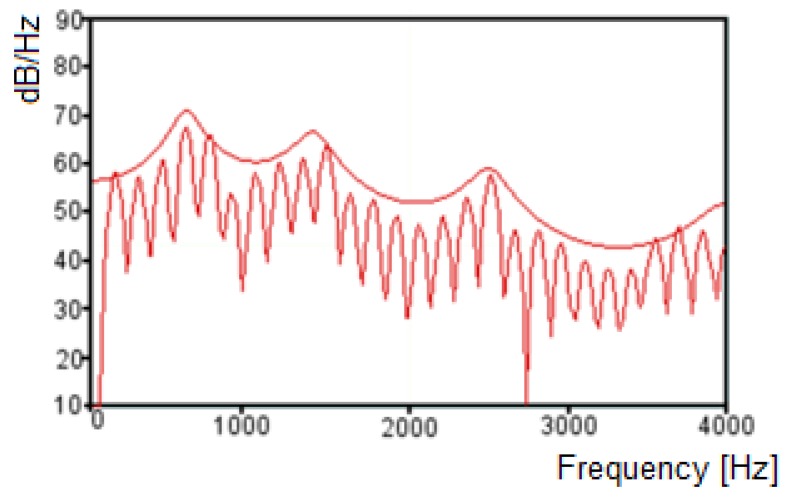
Fourier spectrum and linear predictive coding (LPC) envelope for a given audio signal.

With the roots obtained by the LPC model, it is possible to calculate the formant frequencies, using the tangent arch coordinated on the unitary circle [35,36,37], which reflect the peaks estimated by the LPC model [33]. The criteria commonly used for formant frequencies selection by LPC considers 90 Hz as a minimum frequency, and the bandwidth is less than 400 Hz [26]. Figure 1 presents the spectrum obtained by the decomposition of a given audio signal on its frequency components, and the envelope through LPC. With the information represented on the LPC envelope, it is possible to detect the peaks of frequency, which represent the frequencies that are a candidate to formants, and its bandwidth, the characteristics required to determine the formants.

### 2.3. Least Ordinary Squares Method

The method proposed for audio comparison is based on OLS by taking into account only one explanatory variable. Regression through OLS is a statistic tool that aims to estimate the relationship between a dependent variable and one or more independent variables [38]. In this study, a comparison was performed between two audio segments (reference formant versus confronted formant). Thus, the formula is given considering the equation of a line:

(7) y=α+β∗x,

where *x* is the line point, *y* is the answer variable, β is the angular coefficient, and *α* the linear coefficient. The fitting quality of the linear model is computed by evaluating the fitting’s residual squares. The OLS method is widely used in a number of contexts, such as econometrics, engineering, and data science. The manner in which OLS works is to propose a model that adjusts to the data, so that the sum of the distances’ magnitude between each point in relation to the proposed model is the smallest possible [39]. This is achieved according to the following equations:

(8) $$y_{i}=\alpha +\beta *x_{i}+\varepsilon_{i}$$

(9) $${\hat{\varepsilon }}_{i}=y_{i}-\alpha -\beta *x_{i},$$

where, ${\hat{\varepsilon }}_{i}$ represents the error, and *x_i_* and *y_i_* are the formants for the compared audios. The least squares estimators are given by:

(10) $$y_{médio}=\frac{\left(\sum_{i=0}^{n}y_{i}\right)}{n}$$

(11) $$x_{médio}=\frac{\left(\sum_{i=0}^{n}x_{i}\right)}{n}$$

(12) $$\hat{a}=y_{médio}-\hat{\beta }*x_{médio}$$

(13) $$\hat{\beta }=\frac{\sum_{i=0}^{n}\left(x_{i}-x_{médio}\right)*\left(y_{i}-y_{médio}\right)}{\sum_{i=0}^{n}{\left(x_{i}-x_{médio}\right)}^{2}}=\frac{Cov\left(x,y\right)}{Var\left(x\right)}=r_{xy}*\frac{S_{x}}{S_{y}},$$

where, *r_xy_* is the coefficient of sampling correlation, *s_x_* and *s_y_* are the non-corrected sample standard deviations of *x* and *y*, $\hat{a}$ is the constant regressor term, and $\hat{\beta }$ is the scalar regressor term of a linear model. The determination of the coefficient R-square is given by:

(14) $$R^{2}=r_{xy}^{2}.$$

### 2.4. Statistical Comparison Criteria

With the aim of comparing the generated model statistically, an F-test was performed. This is used to assess the data fitting quality. An F-test computes the statistics over the values of the sums of residual squares on the model tested [40]. Thus, this assumes an arbitrary model (naïve model) constructed from the parameters of the model tested. The F-Test indicates whether the model tested is capable of adjusting itself to the data significantly better than the arbitrary model. The F-test is performed by the following equation:

(15) $$F=\frac{\frac{RSS_{1}-RSS_{2}}{m_{2}-m_{1}}}{\frac{RSS_{2}}{n-m_{2}}},$$

where *RSS* represents the residual squares between the arbitrary model (*m*_1_) and of the model tested (*m*_2_), and n are each of the data points compared amongst the models. Therefore, if the test returns a significant value, it indicates that the linear regression model predicts the response variable better than simply the average of that response (arbitrary model) [40]. The significance values are calculated considering α = 90% and the *p*-Value is represented according to Table 1.

### 2.5. Q–Q Plot 

A Q–Q plot is a tool used to compare two probability distributions [41]. This is shown through a graphical representation of the spread amongst the quantiles of these two distributions, trying to evaluate how a distribution adjusts to another [42]. Thus, if both probability distributions are identical, this dispersion is given as a straight line, where its quantiles must also be identical [41]. However, any deviation from a straight line expresses characteristics that indicate the difference between these two distributions.

**Figure 2:**
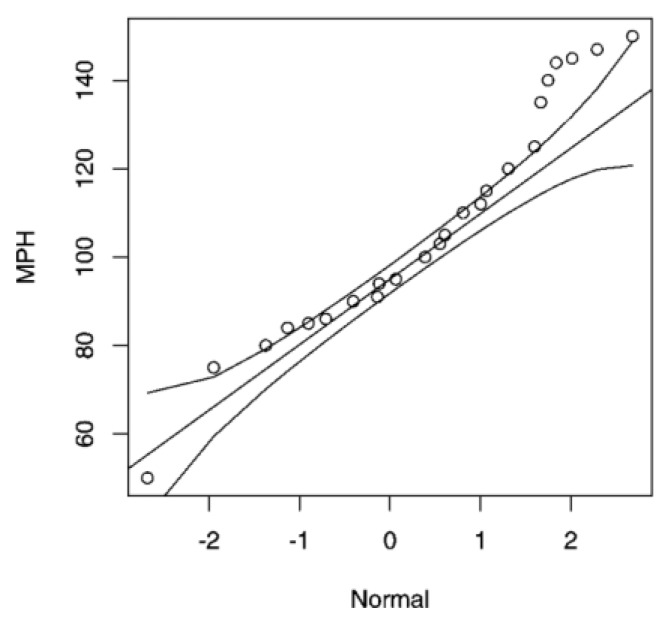
Q-Q Plot for dispersion for a given data regarding a normal distribution.

Using a Q–Q plot helps to compare the adjustments of a data group to the normal distribution, representing the graphic spread of the quantiles of the distribution in question, and the quantiles of a normal theoretical distribution [42]. Besides, it is useful to compare any two given distributions and to assess the extent of any similarity or difference, according to the spread in relation to the straight line. It is also a very useful method for outlier detection in the data group [41]. Figure 2 shows the comparison of dispersion for a given data regarding a normal distribution. The straight line stands out, with the curved lines representing the standard deviation of the distribution on either side.

## 3. Proposed Method 

**Figure 3:**
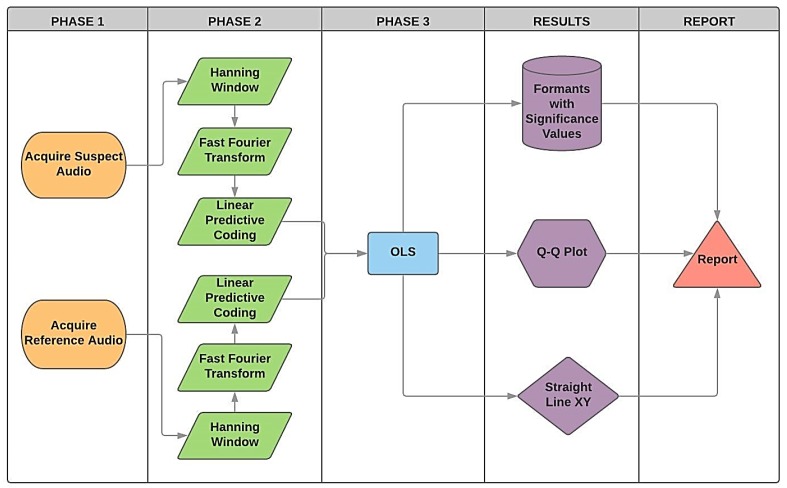
Structure developed for forensic speaker comparison, based on ordinary least squares (OLS), including all three phases.

Figure 3 presents the framework for the proposed method that is expected to produce the result with an aim of confirming whether the confronted audio corresponds to the reference. This approach can be summarised as follows. First, the same sentence produced on the contested audio is recorded by an alleged reference (Phase 1). Next, it performs the windowing followed by the Fourier transform. Posteriorly, the LPC algorithm extracts the formants (Phase 2). In the next step, it uses the OLS model to compare statistically whether each formant presents any degrees of significance (Phase 3). In Phase 4, the Q-Q plot and the XY straight-lines are plotted for each formant on the confrontation amongst both audios. In Phase 5, the methodology supports the production of a final report, that can then be analysed by a forensics expert.

### 3.1. Phase 1: Acquisition of Contested Audios and Reference

Audio files were acquired and captured using a cell phone device in a controlled environment, with the phone positioned next to the speaker. The files were sampled at 48 kHz in stereo audio mode. The individual played the proposed sentences used to perform the comparison with the speaker. The recorded sentences were as follows (in Portuguese):

#1 “O rato roeu a roupa do rei de Roma”;
#2 “O macaco mordeu a macacada no monte Maia”;
#3 “O macaco mordeu o sapato”.

It is important to point out that this study employed short sentences for speaker comparison because this would significantly reduce the error rate. The performances of the automatic speaker verification systems degrade, due to the reduction in the amount of speech used for enrolment and verification. Combining multiple systems (based on different features and classifiers) can considerably reduce the speaker verification error-rate with short utterances [43].

Audio processing was conducted using an application developed in Matlab 2018b. First, data were loaded by a guest user interface (GUI), where the user could select the desired audio file. (The software is able to support any audio file format, such as m4a, ogg, wav, mp3, and mp4). However, the default format .m4a was used here. After loading the audio file, the user could opt to play the selected track to check the file.

### 3.2. Phase 2: Formants Extraction

The audio file was then segmented in smaller parts of adjustable duration, according to the following equation:

(16) $$t_{jan}=\left(\frac{0.45}{pitch_{floor}}\right)*1000,$$

where, *t_jan_* is the window size in seconds, and *pitch_floor_* is the smallest frequency expected for that audio file analysed, selected by the user. The segments have an overlap (Hanning window) to allow for greater continuity amongst the data, and its value can be selected by the user. It is set with a 90% overlap as default. Through Equation (16), the windowing time is kept below 25 ms intervals, which is the minimum amount required for the extraction of spectral characteristics referring to the audio signals [44].

After data segmentation, the decomposition of the frequencies’ power spectrum for each segment was performed. To do so, a Hanning window was applied [28] to smooth the effects of the edge that may distort the signal. Next, the fast Fourier transform (FFT) was computed for the segments. As a result, a new set of data was obtained comprehending the spectrum powers on the interval from 0–24 kHz.

The next step was to estimate the number of coefficients for each data windowed, aiming to extract the pitch and the formant frequencies of the signal via LPC [33]. Then, the frequencies were obtained according to the minimal frequency criteria (according to the windowing stage of the signal) and to the maximum acceptable bandwidth [26]. The frequencies that matched both criteria were considered, the first being pitch *(F0)*, and the subsequent being *F1* to *F4*. In the literature, it was demonstrated that these are liable to encompass the spectral information regarding each vowel [26]. However, the developed software runs on extracting the greatest possible number of formants from audio (approximately 16–20 formants). It is important to mention that the number of formants depends on the spectral properties of the voices of each individual. The automatic extraction of acoustic properties occurred through the LPC technique, which reduced the data collected by linear regression. Then, the obtained data were stored for comparison amongst speakers. A comparison was conducted in pairs through data analysis of the formants via the OLS algorithm. 

### 3.3. Phase 3: OLS and Statistical Comparison

The data were grouped according to each specific formant, and compared via OLS by means of linear regression [39]. The linear model implemented returns the characteristics related to the comparison of each one of the audio formants compared. It makes the resulting assessment by comparing *p*-values that result from the F-test performed by the model. To judge the results obtained by the implemented model, the significance value returned by pitch analysis was tested, which was the first of the criteria. Next, the analysis was completed ensuring formants which helped with the comparison amongst speakers. The pitch comprises the most relevant frequency for the comparison. Increasing the number of auxiliary formants was expected to return significant values by the model producing a greater amount of evidence that the compared voices coming from the same speaker. 

A Q–Q plot was used to corroborate that the residuals of the compared formants had a similar distribution. This showed that they comprised similar frequency ranges, regardless of the result they presented in the model. Finally, the XY line was plotted by confronting each formant of the contested audio with the one of reference. This demonstrated how each data point was related to its respective distance from the XY line of the OLS model.

## 4. Experimental Results

**Figure 4:**
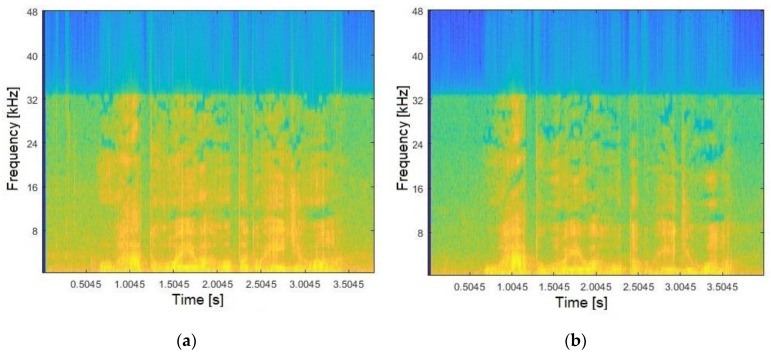
Audio frequency spectrum for: (**a**) confronted; (**b**) reference.

To verify whether a confronted audio corresponds to a reference, the spectrum pattern analysis consisted of the first step. The audios were acquired as presented in Phase 1. Hence, Figure 4a illustrates the frequencies spectrum generated for a given suspect, whilst Figure 4b shows the spectrum for the reference (both relating to Sentence #1). The results were obtained by applying Hanning windowing (90% overlapping) and the FFT (Phase 2). As indicated by Figure 4, analysing audios using only the frequencies spectrum, besides being complex becomes too subjective and tends to introduce error, ultimately requiring more data information for the decision-making process. 

**Figure 5:**
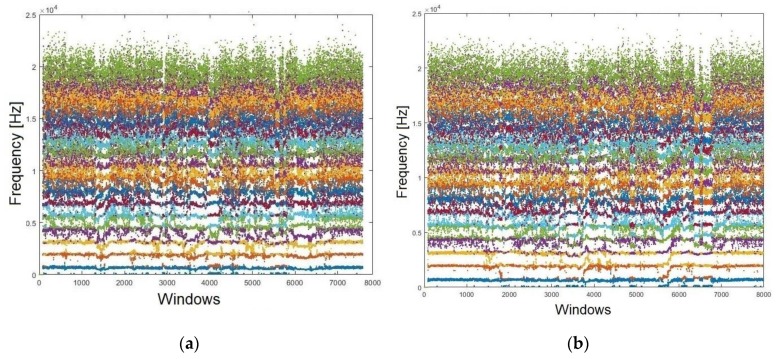
Formants (N = 19) extracted from both audios: (**a**) confronted; (**b**) reference.

It is important to point out that other important factors for our approach are how the formants are positioned, along with the amount of comprise. Accordingly, the LPC algorithm was conducted. Figure 5 shows the behaviour of each formant with its respective frequencies. Figure 5a represents the contested and Figure 5b the reference audios, respectively. These relate to Sentence #1. The 19 obtained formants for each audio are thus presented.

**Figure 6:**
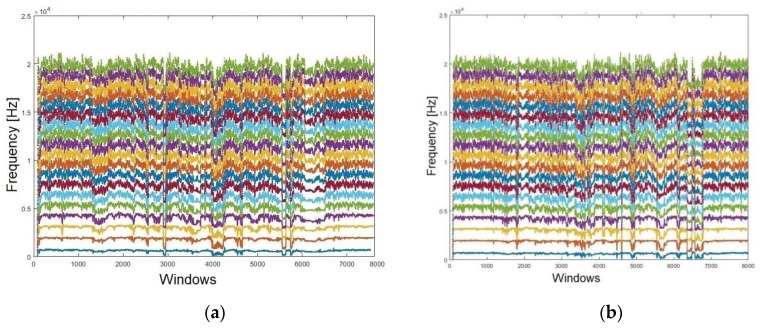
Smoothing the formants using an MAV filter for: (**a**) confronted audio; (**b**) reference audio.

Note that it is still not possible to establish a parameter for a reliable result based only on graphics. To enhance the visualisation, a moving average filter (MAV) was conducted. This filter is used to lighten the dots, rendering them more continuous. Figure 6a shows the formants extracted from the contested audio, whilst Figure 6b presents those extracted from the reference audio, following the MAV application (both relating to Sentence #1). It is notable that certifying the likelihood amongst the formants’ spectrums, even after applying the MAV filter, presents a difficult task. Accordingly, it was necessary to quantify those subtle differences statistically, aiming to achieve a much more accurate analysis as demanded in the forensic context.

To obtain a robust method to verify the speaker, this paper proposed the use of the OLS model, whereby the reference formants were compared to the audio confronted by the OLS model. The small *p*-value resulted in a higher significance. In this approach, the highest significance was represented by ‘***’. It is important to highlight that pitch is the main formant, and if it presents no ‘***’, the comparison is considered as negative, requiring the remaining formants to present at least one ‘*’. Accordingly, it must not accept non-significant (NS) for the analysed sentence. Hence, Table 2 presents the results obtained using OLS comparing the confronted audio with the reference (both for Sentence #1). After analysing the results, it is evident that the 19 formants showed high significance levels. The smaller the *p*-value, the stronger the correlation amongst the pairs (formant *F3*). 

### 4.1. Validating the Model

**Figure 7:**
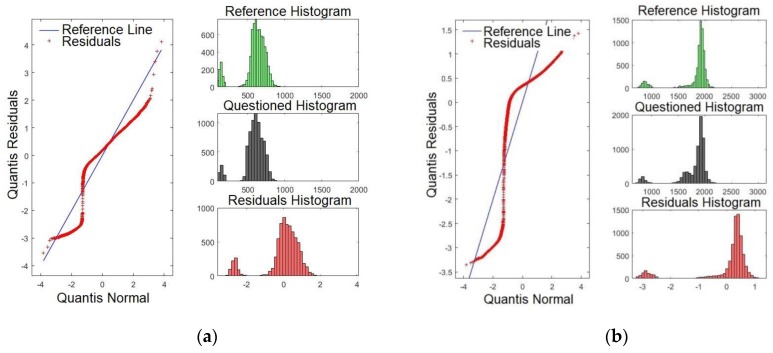
Q-Q plot of the formants comparing the confronted with the reference audios: (**a**) formant *F0* and (**b**) formant *F1*.

To compare normal distributions, the Q–Q plot can be utilised. The aim was to validate whether the model presents a similar distribution by verifying whether the histogram of the reference was close to that of the confronted audio. If an affirmative result is achieved, this provides a positive result for the speaker comparison, ensuring that the normal distribution is confronted with the same audio segment. The residual histograms, in turn, are inversely proportional to the quality of the formant. This means that smaller matches of histograms lead to a better *p*-value, increasing the reliability of the model. For the purpose of brevity, only some formants are shown. Hence, Figure 7a,b shows the Q–Q plots for formants *F0* (pitch) and *F1*, respectively. Henceforth, all figures presented in this paper relate to Sentence #1. By examining Figure 7a, it is evident that there are four intersection points of the residues with the straight line: one high, two medium, and one low intensity. Conversely, Figure 7b presents two intersection points, one high and one low intensity. To conclude, *F0* presents a higher degree of significance compared with *F1*, as previously presented in Table 2 (*p*-value).

**Figure 8:**
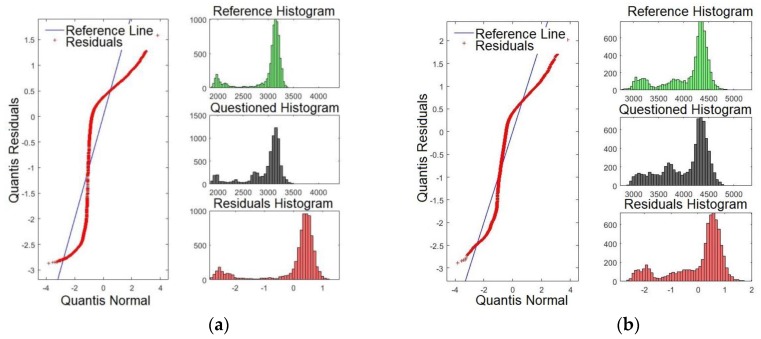
Q-Q plot of the formants comparing the confronted with the reference audios: (**a**) formant *F2* and (**b**) formant *F3*.

Figure 8a,b shows the Q-Q plots for formants *F2* and *F3*, respectively. By examining Figure 8a, it is evident that there are three points where the residues intersect with the straight line: one is very high and two of high intensity. Conversely, Figure 8b presents three high intersections. This result matches with those in Table 2, where *F3* was shown to present the higher *p*-value.

**Figure 9:**
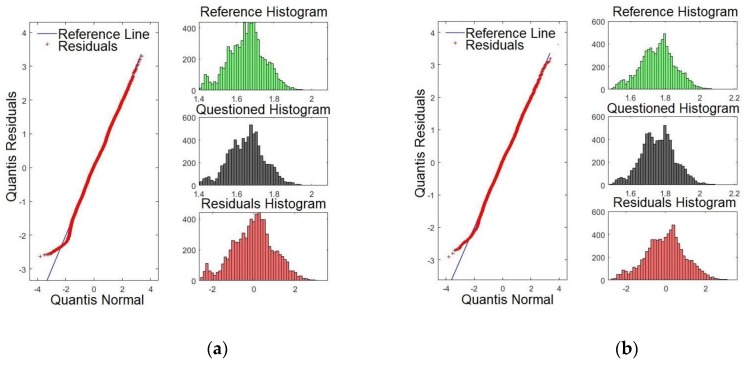
Q-Q plot of the formants comparing the confronted with the reference audios: (**a**) formant *F15* and (**b**) formant *F16*.

Figure 9a,b presents the Q-Q plots for formants *F15* and *F16*, respectively. By examining Figure 9a,b, it is evident that the line of the residues coincides with the reference line, showing that the significances are weaker compared with those presented for *F3*, for example. These results match with those presented in Table 2, where *F15* and *F16* presented smaller *p*-values.

**Figure 10:**
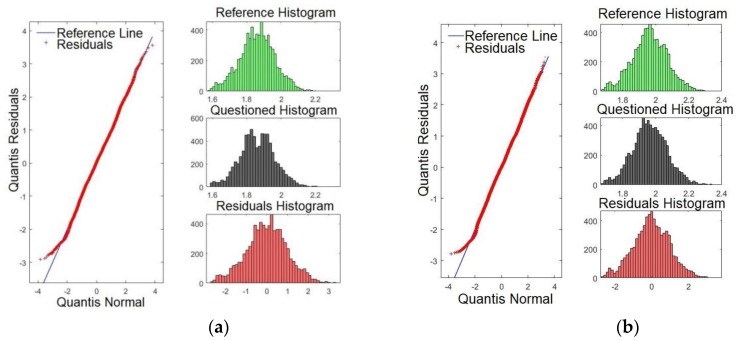
Q-Q plot of the formants comparing the confronted with the reference audios: (**a**) formant *F17* and (**b**) formant *F18*.

Figure 10a,b shows the Q-Q plots for formants *F17* and *F18*, respectively. Likewise, the results indicate that the line of the residues coincides with the reference line, showing that significances are weaker than for *F2* and *F3*, for example. These results match with those presented in Table 2. There, *F17* and *F18* presented the smallest *p*-values. It is expected that the last four formants have less satisfactory results compared with the first formants. However, they may be important to verify speakers with similar voice tones.

It is important to point out that although some formants on the Q–Q plot are almost coincident with the straight line, this does not disqualify the results of the model. The Q–Q plot is a comparison of how the data adjusts in relation to the norm. Thus, divergences regarding the central line indicate that there is a difference in the frequency regime of the compared formants, but does not suggest problems with the quality of the model. Besides, the similarity on the profile of residues indicates that the formants have similar patterns. However, the comparison through a linear model may (or may not) return a significant value, regardless. Thus, the Q–Q plot shows that the compared frequency strip amongst formants is similar which reinforces the formants extracted method by using the LPC.

**Figure 11:**
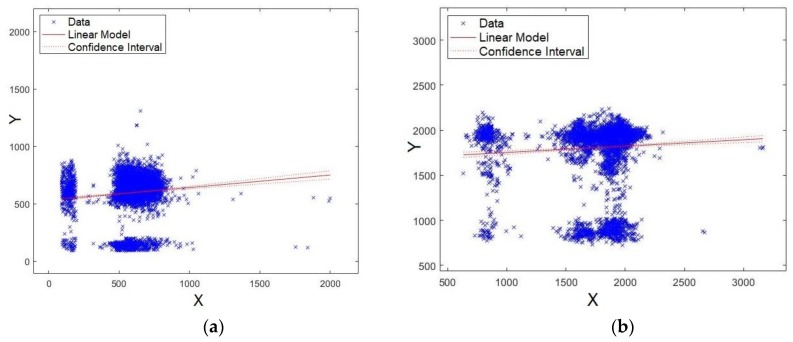
XY straight line for: (**a**) formant *F0* and (**b**) formant *F1*.

Here, the obtained model via an XY plot was also evaluated. It was expected that the data would be close to the straight line, which would indicate a good fit for the model. As a consequence, it is implied that the level of significance of the formant is stronger. Figure 11a,b shows the plots for formants *F0* and *F1*, respectively. As observed from the figures, the data are agglomerated close to the straight line, showing that the models for formants *F0* and *F1* have strong significance. 

**Figure 12:**
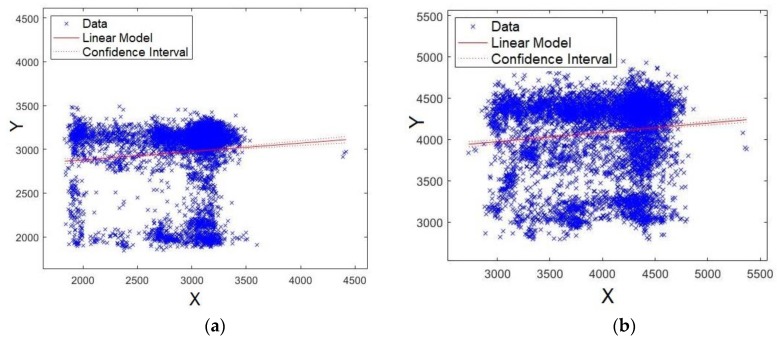
XY straight line for: (**a**) formant *F2* and (**b**) formant *F3*.

Coincidently, Figure 12a,b shows the plots for formants *F2* and *F3*, respectively. As observed, once again, the data are agglomerated close to the straight line, showing that models for formants *F2* and *F3* have strong significance. 

**Figure 13:**
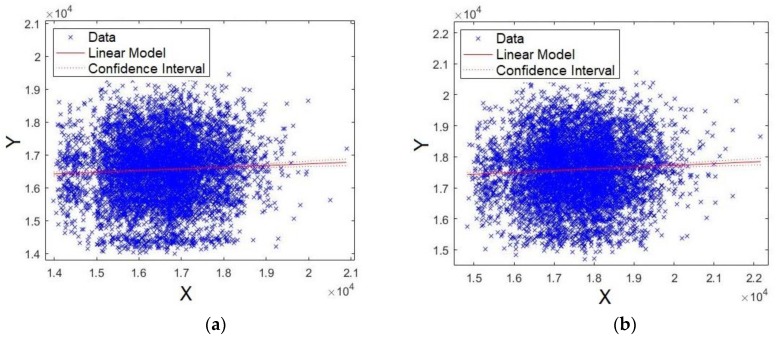
XY straight-line for: (**a**) formant *F15* and (**b**) formant *F16*.

**Figure 14:**
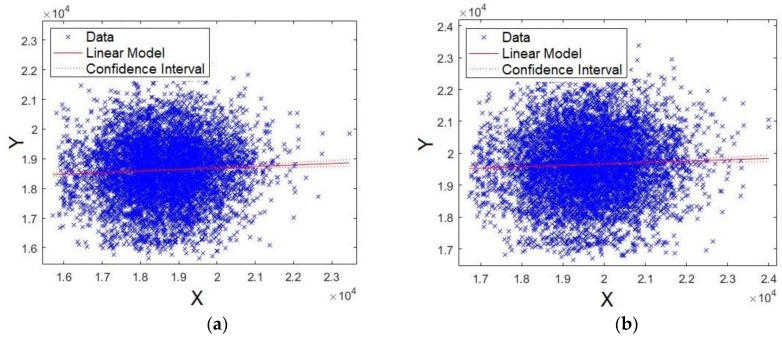
XY straight line for: (**a**) formant *F17* and (**b**) formant *F18*.

The last four formants were also evaluated. First, Figure 13a,b shows the plots for formants *F15* and *F16*, respectively. Unlike the results already presented, the data are scattered around the straight line, showing that models for formants *F15* and *F16* have lower significance compared with *F0*, *F1*, *F2*, and *F3* (Figure 11 and Figure 12). Similar results were also shown for *p*-values and Q–Q plot. Careful attention should be given to the scales of axes for Figure 13 and Figure 14, because they are multiplied by 10^4, unlike Figure 11 and Figure 12.

Second, Figure 14a,b shows the plots for formants *F17* and *F18*, respectively. As observed from these figures, the data are scattered around the straight line, showing that the models for formants *F17* and *F18* present lower significance compared with *F0*, *F1*, *F2*, and *F3* (Figure 11 and Figure 12). Similar results have already been shown for *p*-values and Q–Q plots. 

### 4.2. Practical Results

To validate the proposed method, practical tests were conducted considering a database composed of 26 speakers (13 males and 13 females), where all 26 as suspects were classified ordinally (see Table 3). Another audio was recorded from one of these and this was taken as the reference. The column “Time” refers to the length of the recorded audio. An interesting scenario was performed, whereby the suspects spoke Sentence #2 (as the reference audio), and the contested audio from Sentence #3 was recorded. Table 3 shows the results for *p*-values comparing all suspects and formants.

From Table 3, it is evident that suspect #3 attained a higher significance value of ‘***’ and only one formant, NS. These results are in an acceptable level of confidence to certify positively that suspect #3 is the speaker responsible, according to the contested audio. By analysing Table 3, it might be considered that suspect #11 could be charged as the speaker. It is important to highlight that the significance value returned by pitch analysis (*F0*) must be taken into account at the first criterion of analysis. As the *p*-value is NS for *F0*, this hypothesis should be rejected. This test also took into account a sentence with suppression and change of words, as well as the peak of frequencies being different for each character narrated. This analysis allows the validation of the developed model, leading to the verification of which speaker is responsible, even under some interference in the recorded audio. 

Table 4 shows the comparison for another scenario, whereby Sentence #1 is recorded for both the contested and reference audios. The comparison confronted several suspects, including some of those previously used in Table 3. Once again, suspect #3 achieved maximum significance in all formants (a positive result), validating the hypothesis that the confronted audio belongs to that suspect.

Table 5 presents a new scenario. The idea was to investigate the correlation of the reference audio for Sentence #1 with itself. It was highly anticipated that all formants would be positively identified, demonstrating then that the audios were identical. As shown in Table 5, the *p*-values are 0 for all formants. This clearly demonstrates that both compared audios are equal, showing that the proposed model can also detect with accuracy in this scenario.

Posteriorly, it was proposed to verify the effect of the quality of audios on the proposed method, with the results presented in Table 6. First, it is important to highlight that 128 kbps is the standard quality. Here, this study compared contested audios at low (64 kbps), medium (128 kbps), and high (256 kbps) qualities with the reference at the standard quality (128 kbps). The tests were conducted for suspect #3. By examining Table 6, it is evident that the audios sampled at the same frequencies tend to obtain better results. However, 19 formants were extracted. Conversely, only 16 formants were obtained when considering a higher quality for the contested audio (256 kbps). Despite this point, the positive results verify the speaker. On the contrary, low-quality audio (64 kbps) for the contested audio, decreased the number of formants to 17. Although some non-significative *p*-values were obtained, the high number of “***” ensured that the speaker was positively identified.

Another fundamental test consists of investigating whether different audio timing has influence on the results of the developed model. Accordingly, the timing of the reference audio was noted as very slow, slow, and very fast. The results are shown in Table 7. The test was also carried out for suspect #3, considering the length of time of the contested audio (3.817347 s) for Sentence #1. 

From Table 7, it is evident that the *p*-values have reduced significantly. First, it should be noted that formant *F18* was not recognised. Second, if the audio time is played in slow motion, the level of significance diminishes for the last formants. However, the result is still positive for speaker verification. Conversely, when the audio speeds up, the level of significance of the formants oscillates, where even *F1* gets NS. However, the results still positively verify the speaker.

Finally, the different levels of noise on the contested audio were examined followed by the statistical confrontation. The test was also carried out for suspect #3, considering brown, pink, and white noise, for Sentence #1. This represents 50%, 5%, and 1% of the spectrum. These results are presented in Table 8. First, by examining Table 8, it is remarkable that after noise insertion, the maximum number of formants was 10. Despite that, the results could positively verify speaker identity, given that all other *p*-values were “***”. Overall, all seven experimental tests hit 100% accuracy, demonstrating that the method is well suited to verify the speaker in a forensic context.

To evaluate real scenarios and to better validate the method, other audios containing various conditions (reference audios) were tested. These records were obtained after more than 30 days from the contested audio, all for Sentence #1. Table 9 depicts the results obtained. First, a telephone interception was carried out. Accordingly, the phone call was carried out on the street considering a noisy scenario (cars, wind and people’s voices). Owing to the phone channel limitation, only 5 formants were possible to be obtained. After applying the proposed method, it achieved good significance in all formants (a positive result), validating the hypothesis that the confronted audio belongs to that suspect. On another day, a WhatsApp message was recorded (at home). In this scenario, children playing and TV background noise were present. Once again, it results in a positive match. Notwithstanding, a new audio record was performed in a restaurant during a birthday celebration (Sony recorder Icd-PX470). From the analysis, 18 formants were obtained. As shown in Table 9, this clearly demonstrates that the proposed method may also detect with high accuracy in this scenario. Afterwards, another record was carried out in an office by using the computer microphone. There was also background noise caused by the air conditioning, mouse and, keyboard. As afore obtained, the results ensure that the speaker is positively identified. It is important to point out that the number of formants is intrinsically limited to the type of audio recorder used. Apart from this, the results demonstrated that the method is well suited to verify the speaker in several scenarios containing noise and for different recording devices. 

Finally, the proposed method was preliminarily evaluated utilizing the dataset LibriSpeech [45]. This dataset is a public domain and contains 1983 speakers. All of them spoke in English. They conducted all the recordings for months, with different accents, mixing female and male, etc. First of all, the file named as 9023-296467 was considered as questioned audio. Accordingly, 20 different audios were considered as the references (Table 10). Based on the proposed methodology, this study observed that audios named as 9023-296468 and 7177-258977 are the best candidates to be the speaker. The 9023-296468 speaker attained a higher significance value of ‘***’ and only one formant NS, being the correspondent speaker for the test (positive verification). It is important to highlight that those audios for 9023-296468 and 9023-296467 were recorded by the same speaker at a different time. For further details about the dataset formation, readers can explore the following reference [45]. However, readers should pay attention to the best *p*-value (F0) that was accomplished for speaker #9 (10^−7^), which may be considered a poor value given an unnoisy condition. Comparing it with Table 2, whereby 10^−22^ was obtained for the best *p*-value, English may be considered as a non-adaptive language for the proposed method, since if it inserts noise, all *p*-values decrease to a non-significant value in several formants. The reason might be explained back to the history of the Portuguese language which emerged in the thirteenth century, undergoing a Latin evolution. The Brazilian Portuguese language has a complex vocabulary, having a symmetrical and balanced phonetic system with the final notes more clearly than in European Portuguese [46]. On the other hand, the history of the English language begins in the year 499 BC, 5th Century. Accordingly, it is much older than the Portuguese language and, since then, has had influences from the Anglo-Saxons and later from the French. In this context, the vowel sound system has changed substantially from the correlation between spelling and pronunciation, distancing itself from other Western European languages [47]. Therefore, the results here reached are legitimate for Brazilian Portuguese and these strongly reinforce the potentiality of this work, since the several voices dataset are in English which could provide distance from Brazilian Portuguese. In the same way, several methods found in the literature, for speaker identification, might be not work accurately for Brazilian Portuguese. 

## 5. Comparison with Other State-Of-The-Art Solutions

Praat is an open-source software package used for the analysis of acoustic parameters, such as formants. This software is used comprehensively in linguistic and forensic applications worldwide. It can perform speaker comparison by using a graphic comparison of spectra and absolute values. Its main disadvantage is low accuracy, hampering its usage at a forensic level. Notwithstanding this, the methodology for speaker comparison developed here, based on extracting formants via LPC followed by applying the OLS algorithm, is novel in the forensic literature. 

To evaluate the proposed method, Table 11 depicts a comparison between the proposed method and Praat in terms of execution time, number formants, lines, and supported formats. It is important to point out that Praat only extracts formants, whilst the proposed method does this and statistically evaluates the model to provide an accurate result for forensics experts. Examining Table 11, it can be verified that the runtime to extract the formants is a time-consuming task for the proposed method compared with Praat software. This can be explained if the number of formants extracted by the proposed method is analysed, which is almost four times as many. Likewise, the number of lines is approximately seven times greater. Conversely, the proposed method is more robust, leading to a much more reliable result. Once again, it is important to highlight that this time comparison is only for the purpose of generating the formants and part of the graphs to the same extent that the Praat software can provide. 

Overall, the total time to insert data, select the desired options, extract the formants, perform the OLS, compare the audios, and generate all graphs can take up to 112 s, which is fast, as it performs countless mathematical tasks and plots approximately 44 graphs. While Praat software supports seven audio formats, the developed method supports all audio files, providing a meaningful advantage, even where the contested audio is recorded by a unit where it needs to export to a different audio format. In this case, it would be necessary to convert the audio to some Praat supported format. This could result in data loss and/or smoothing peaks of the formants, which would be strictly inadmissible in a forensic context.

## 6. Final Remarks

This paper has presented an exploration of the suitability of an LPC-OLS based method applied to the verification of speaker identity in a forensic context. To date, there is no evidence of any another method that focuses on the same issues presented here. Besides the fact that the LPC-OLS based method is proposed here for the first time, the major contribution of this approach comprises a reliable method to extract more effective formants, followed by statistical testing of the model (using OLS) to provide an accurate result for the forensics expert.

As a result, the proposed method was able to verify the speaker successfully with a higher accuracy rate when using the LPC-OLS based method. It is important to highlight that after running seven different tests, the method yielded a 100% accuracy rate (which included suppression and word changes, a sentence without adjustments, different qualities on the audio, and uneven speech speeds and noise levels). Nevertheless, it is important to highlight that the main contribution is to Brazilian Portuguese in which this approach showed as successful after being preliminarily validated with a limited database and, it can only be considered as a study direction since they were limited to a few scenarios. Furthermore, the results strongly reinforce the potentiality of this work, since the several voices dataset are in English which could provide distance from Brazilian Portuguese. In the same way, several methods found in the literature for speaker verification, might be not work accurately for Brazilian Portuguese. 

Comparing the method with Praat software, it can clearly be observed that the proposed approach presents a higher number of formants, which reduces the chances of false-positives when performing speaker comparison. Aside from that, the high number of lines used to extract the formants produces a significant matrix of incremental robustness for the proposed model. Unlike Praat software, the developed method supports all audio formats which may prevent data loss or smoothing the formant peaks during a given format conversion. In summary, the outcomes of this study have shown the efficiency, accuracy, and robustness of the proposed method in detecting speaker identity, considering the studies found in the forensic literature. 

Despite the advantages, improvements of the proposed method still need to be investigated. Hence, future work can be completed to evaluate the sensitivity of the developed method to extract more formants. For example, this might consider other levels of noise and different audio qualities. In addition, much more research is expected to be conducted on evaluating formant comparison via OLS by considering increasing the size of the dataset and optimising time-consumption. Furthermore, future research can be undertaken to form a database for the Brazilian Portuguese speaker containing audios recorded after different time periods, under various acoustic conditions, for each ethnic group, the sex and age range, noise and various recording devices. 

## Figures and Tables

**Table 1:** Intervals for determination of *p*-Value for the F-test.

Symbol	*p*-Value
**‘NS’** (non-significant)	*p* > 0.1
‘*’	0.05 < *p* ≤ 0.1
‘**’	0.01 < *p* ≤ 0.05
‘***’	*p* ≤ 0.01

**Table 2:** Significance levels obtained by the OLS model considering the analysed audios.

Row	*p*-Value	Significance
Pitch (F0)	4.45191E-17	***
F1	3.0643E-08	***
F2	5.21042E-15	***
F3	3.28572E-22	***
F4	8.03937E-11	***
F5	1.63395E-11	***
F6	9.19947E-14	***
F7	8.38252E-14	***
F8	2.15671E-10	***
F9	1.75295E-08	***
F10	7.46944E-09	***
F11	6.53178E-08	***
F12	7.18641E-07	***
F13	2.64407E-07	***
F14	2.29227E-06	***
F15	1.23279E-05	***
F16	1.24595E-06	***
F17	1.37544E-05	***
F18	0.000179185	***

**Table 3:** The results of the comparison between the suspects (Sentence #3) and reference (Sentence #2).

Suspect	Time(s)	Pitch	F1	F2	F3	F4	F5	F6	F7	F8	F9	F10	F11	F12	F13	F14	F15	F16	F17	F18
1	4.52644	NS	***	***	NS	NS	NS	NS	NS	NS	NS	NS	NS	NS	NS	NS	NS	NS		
2	5.715986	*	***	**	NS	NS	NS	NS	NS	NS	NS	NS	NS	NS	NS	NS	NS	NS	*	NS
3	4.5	***	***	NS	*	***	***	***	***	***	***	***	***	***	***	***	***	***	***	
4	4.457347	***	***	***	NS	NS	NS	NS	NS	*	NS	NS	NS	NS	NS	NS	NS	NS	NS	NS
5	2.942653	***	***	**	NS	NS	NS	NS	NS	NS	NS	NS	NS	NS	NS	NS	NS	NS	NS	NS
6	4.244014	***	***	***	***	***	***	***	***	*	NS	NS	NS	NS	NS	NS	NS	NS	NS	NS
7	4.265351	NS	NS	NS	NS	NS	NS	NS	NS	NS	NS	NS	NS	NS	NS	NS	NS	NS	NS	NS
8	3.732018	***	NS	*	NS	NS	NS	NS	**	**	**	**	**	***	***	***	**	*	*	**
9	3.092018	***	***	NS	NS	NS	NS	NS	NS	NS	NS	NS	NS	NS	NS	NS	NS	NS	NS	NS
10	4.154921	NS	NS	NS	*	NS	NS	NS	NS	*	NS	NS	NS	NS	NS	NS	NS			
11	4.03068	NS	*	***	***	***	***	***	***	***	***	***	***	***	***	***	***	***	***	***
12	5.118685	NS	***	NS	*	***	***	***	***	***	**	***	**	**	**	**	*	**	*	NS
13	3.412018	NS	NS	***	***	***	***	***	**	**	*	*	*	NS	NS	NS	NS	NS	NS	NS
14	3.966667	NS	NS	NS	*	NS	NS	NS	NS	NS	NS	NS	NS	NS	NS	NS	NS	NS	NS	NS
15	3.433333	***	*	NS	NS	NS	NS	**	**	NS	*	NS	NS	NS	NS	NS	NS	NS	NS	NS
16	3.54	**	***	***	***	***	***	***	***	**	**	**	***	**	*	NS	NS	NS	NS	NS
17	4.99068	*	NS	*	NS	*	NS	NS	NS	NS	NS	NS	NS	NS	NS	NS	NS	NS	NS	
18	4.18	*	***	***	***	***	**	*	NS	NS	NS	NS	NS	NS	NS	NS	NS	NS	NS	NS
19	4.5	***	***	***	***	**	***	NS	NS	NS	NS	NS	NS	NS	NS	NS	NS	NS	NS	NS

**Table 4:** The results of the confrontation between the suspects (Sentence #1) and reference (Sentence #1).

Suspect	Time(s)	Pitch	F1	F2	F3	F4	F5	F6	F7	F8	F9	F10	F11	F12	F13	F14	F15	F16	F17	F18
3	3.736961	***	***	***	***	***	***	***	***	***	***	***	***	***	***	***	***	***	***	***
4	2.686667	***	***	**	NS	*	NS	NS	NS	*	**	**	**	***	***	***	***	***	**	**
6	3.795986	***	***	***	***	***	***	***	***	***	**	**	***	***	***	**	*	NS	NS	NS
10	4.64254	NS	*	NS	NS	NS	NS	NS	NS	NS	NS	NS	NS	NS	*	*	**	**		
12	4.265351	***	***	NS	NS	NS	NS	NS	*	NS	**	*	NS	NS	**	NS	*	*	**	*
16	3.582653	***	***	NS	NS	NS	**	**	***	***	***	**	**	**	*	*	NS	*	NS	NS
17	3.86	***	*	***	***	***	*	**	NS	NS	NS	NS	NS	NS	NS	NS	NS	NS	NS	NS
18	3.54	NS	*	***	NS	NS	***	*	NS	NS	NS	NS	NS	NS	NS	NS	NS	NS	NS	NS
19	3.22	NS	NS	***	***	NS	*	NS	NS	NS	NS	NS	NS	NS	NS	NS	NS	NS	NS	NS
20	5.097347	***	***	**	NS	**	NS	NS	NS	NS	NS	NS	NS	*	*	NS	NS	*	NS	NS
21	2.857347	***	***	***	NS	NS	NS	*	NS	*	**	**	**	NS	NS	NS	NS	NS	NS	NS
22	2.644014	***	***	***	***	**	**	NS	NS	NS	NS	NS	NS	NS	NS	*	**	*	**	*
23	4.03068	**	***	*	NS	NS	NS	NS	**	**	**	*	*	**	*	NS	*	NS	NS	NS
24	3.412018	***	***	NS	**	**	NS	NS	NS	NS	NS	NS	NS	NS	NS	NS	NS	NS	NS	NS
25	3.113333	NS	NS	NS	NS	NS	NS	NS	NS	NS	**	***	**	**	**	**	*	NS	NS	NS
26	2.835986	***	*	**	NS	*	NS	NS	NS	NS	NS	NS	NS	NS	NS	NS	NS	NS	NS	NS

**Table 5:** The results of the correlation between the reference audio, for Sentence #1, with itself.

	Time(s)	Pitch	F1	F2	F3	F4	F5	F6	F7	F8	F9	F10	F11	F12	F13	F14	F15	F16	F17	F18
p-valor		0	0	0	0	0	0	0	0	0	0	0	0	0	0	0	0	0	0	0
	3.736961	***	***	***	***	***	***	***	***	***	***	***	***	***	***	***	***	***	***	***

**Table 6:** The results of the confrontation considering the different qualities of audios (Sentence #1 for suspect #3).

Quality	Time(s)	Pitch	F1	F2	F3	F4	F5	F6	F7	F8	F9	F10	F11	F12	F13	F14	F15	F16	F17	F18
256kbps	3.9	***	***	***	***	***	***	***	***	***	***	***	***	**	**	***	**			
128kbps	3.817347	***	***	***	***	***	***	***	***	***	***	***	***	***	***	***	***	***	***	***
64kbps	3.752185	***	***	***	***	***	***	***	***	***	**	**	**	*	*	NS	NS	NS		

**Table 7:** The results for the reference, under different timing, with the contested audio with 3.817347s (Sentence #1 for suspect #3).

Speed	Time(s)	Pitch	F1	F2	F3	F4	F5	F6	F7	F8	F9	F10	F11	F12	F13	F14	F15	F16	F17	F18
VerySlow	12.414671	***	***	***	***	***	***	***	***	***	***	***	***	***	***	**	**	**	**	
Slow	5.417347	***	***	***	***	***	***	***	***	***	***	***	***	***	***	***	***	***	***	
VeryFast	2.708005	***	NS	**	***	***	***	***	***	***	***	***	***	***	***	***	***	**	**	

**Table 8:** The result of audios confrontation undergone with insertion of different levels of noise (Sentence #1 for suspect #3).

Noise	Time(s)	Pitch	F1	F2	F3	F4	F5	F6	F7	F8	F9	F10	F11	F12	F13	F14	F15	F16	F17	F18
Brown50	3.817347	***	***	***	***	***	***	***	***	***	***									
Pink5	3.817347	***	***	***	***	***	***	***	***	***	***									
White1	3.817347	***	***	***	***	***	***	***	***	***	***									

**Table 9:** The results of the confrontation between the suspects (Sentence #1) and, reference (Sentence #1) recorded after 30 days, considering noise and various recorder devices.

Conditions	Time(s)	Pitch	F1	F2	F3	F4	F5	F6	F7	F8	F9	F10	F11	F12	F13	F14	F15	F16	F17	F18
PhoneStreet	3.646	***	***	***	NS	***														
WhatsAppHome	3.284	***	***	***	*	**	***	**	***	***	***	***	***	**	***	**	**	**		
Sony_Restaurant	3.903	***	***	***	***	***	***	***	***	***	***	***	***	***	***	***	***	***	***	***
ComputerOffice	4.139	***	***	***	***	***	***	**	***	***	***	***	**	**	**	**	**	**	**	

**Table 10:** The results obtained after applying the proposed method to the dataset LibriSpeech.

Test #	Audio Name	Time(s)	*p*-value for F0	Pitch	F1	F2	F3	F4
1	9023-296468	11.670	3.90716 × 10^−6^	***	NS	***	***	**
2	7859-102518	12.520	0.502099189	NS	**	NS	NS	NS
3	7720-105167	10.160	0.014028753	**	NS	NS	***	**
4	8447-284436	9.980	0.00285753	***	***	**	NS	NS
5	14-212	13.570	0.225905533	NS	NS	NS	NS	NS
6	3906-184005	8.620	0.596373964	NS	NS	*	NS	NS
7	4044-9010	9.040	0.563113699	NS	**	**	*	**
8	6300-39660	11.070	0.049729008	**	NS	NS	NS	NS
9	7177-258977	12.730	1.64337 × 10^−7^	***	**	**	NS	NS
10	8262-279161	10.980	0.004817798	***	NS	NS	NS	NS
11	9000-282380	11.250	0.246992694	NS	***	***	**	***
12	152-87733	12.950	0.347284969	NS	NS	NS	*	*
13	218-131205	12.730	0.482158941	NS	NS	NS	NS	NS
14	398-123602	11.920	0.13380562	NS	NS	NS	NS	NS
15	444-138076	11.170	0.66022574	NS	NS	NS	NS	
16	511-131226	11.040	0.033288607	**	***	*	NS	NS
17	639-124526	12.450	0.255417428	NS	NS	NS	NS	NS
18	766-127193	10.980	0.891719784	NS	***	NS	NS	NS
19	850-131003	11.140	0.001656815	***	NS	**	NS	NS
20	949-134657	9.460	0.080615174	*	*	NS	NS	NS

**Table 11:** Comparison of extracting the formants for the proposed method with the Praat software.

Methods	Execution Time (s)	Number Formants	Lines	Supported Formats
Present Method	27.557	19	7590	ALL AUDIO FILES
PRAAT	0.1	5	1193	AIFC, AIFF, FLAC, NEXT/SUN, NIST, MP3 and WAV

## References

[1] Juang B.H., Furui S. (2000). Automatic recognition and understanding of spoken language—A first step toward natural human-machine communication. Proc. IEEE.

[2] Pegoraro T.F. (2000). Algoritmos Robustos de Reconhecimento de voz Aplicados a Verificação de Locutor. http://www.repositorio.unicamp.br/handle/REPOSIP/259689.

[3] Abdel-Hamid O., Mohamed A., Jiang H., Deng L., Penn G., Yu D. (2014). Convolutional Neural Networks for Speech Recognition. IEEE/ACM Trans. Audio Speech Lang. Process..

[4] Ali H., Ahmad N., Zhou X., Iqbal K., Ali S.M. (2014). DWT features performance analysis for automatic speech recognition of Urdu. SpringerPlus.

[5] Furui S. (2010). Speaker Recognition in Smart Environments. Human-Centric Interfaces for Ambient Intelligence.

[6] Braid A.C.M. (2003). Fonética Forense.

[7] Chou W., Recchione M.C., Zhou Q. (2002). Automatic Speech/Speaker Recognition over Digital Wireless Channels. U.S. Patent.

[8] Ajili M., Bonastre J.F., Kheder W.B., Rossato S., Kahn J. Homogeneity Measure Impact on Target and Non-target Trials in Forensic Voice Comparison. Proceedings of the INTERSPEECH 2017.

[9] Koval S. Formants matching as a robust method for forensic speaker identification. Proceedings of the International Conference on Speech and Computer (SPECOM).

[10] Rodman R., McAllister D., Bitzer D., Cepeda L., Abbitt P. (2002). Forensic speaker identification based on spectral moments. Forensic Linguist..

[11] Becker T., Jessen M., Grigoras C. Forensic speaker verification using formant features and Gaussian mixture models. Proceedings of the Annual Conference of the International Speech Communication Association, INTERSPEECH 2008.

[12] Leuzzi F., Tessitore G., Delfino S., Fusco C., Gneo M., Zambonini G., Ferilli S. A statistical approach to speaker identification in forensic phonetics field. Proceedings of the International Workshop on New Frontiers in Mining Complex Patterns.

[13] Gold E., Hughes V. Front-end approaches to the issue of correlations in forensic speaker comparison. Proceedings of the 18th International Congress of Phonetic Sciences (ICPhS).

[14] Bulgakova E.V., Sholokhov A.V. (2016). Semi-automatic speaker verification system. Sci. Tech. J. Inf. Technol. Mech. Opt..

[15] Irum A., Salman A. (2019). Speaker Verification Using Deep Neural Networks: A Review. Int. J. Mach. Learn. Comput..

[16] Devi J.S. (2019). Language and Text Independent Speaker Recognition System using Artificial Neural Networks and Fuzzy Logic. Int. J. Recent Technol. Eng..

[17] Chung J.S., Nagrani A., Zisserman A. VoxCeleb2: Deep Speaker Recognition. Proceedings of the INTERSPEECH 2018.

[18] Dhakal P., Damacharla P., Javaid A.Y., Devabhaktuni V. (2019). A Near Real-Time Automatic Speaker Recognition Architecture for Voice-Based User Interface. Mach. Learn. Knowl. Extr..

[19] Gujarati D.N., Porter D.C. (2008). Econometria Básica.

[20] Rituerto-González E., Mínguez-Sánchez A., Gallardo-Antolín A., Peláez-Moreno C. (2019). Data Augmentation for Speaker Identification under Stress Conditions to Combat Gender-Based Violence. Appl. Sci..

[21] Smith H.M.J., Baguley T.S., Robson J., Dunn A.K., Stacey P.C. (2019). Forensic voice discrimination by lay listeners: The effect of speech type and background noise on performance. Appl. Cogn. Psychol..

[22] Krobba A., Debyeche M., Selouani S.A. (2019). Multitaper chirp group delay Hilbert envelope coefficients for robust speaker verification. Multimed. Tools Appl..

[23] Rahman M. (2011). Applications of Fourier Transforms to Generalized Functions.

[24] Bailey D.H., Swarztrauber P.N. (1994). A fast method for the numerical evaluation of continuous Fourier and Laplace transforms. SIAM J. Sci. Comput..

[25] Oppenheim A.V., Buck J.R., Schafer R.W. (2009). Discrete-Time Signal Processing.

[26] Leme A.L.M., Marcelino M.A., Prado P.P.L.D. (2016). Margens de tolerância e valores de referência para os formantes de vogais orais para uso em terapias de voz para surdos em computador comercial. CoDAS.

[27] Prabhu K.M.M. (2013). Window Functions and Their Applications in Signal Processing.

[28] Essenwanger O.M. (1986). Elements of Statistical Analysis.

[29] Aparna R., Chithra P.L. Role of Windowing Techniques in Speech Signal Processing for Enhanced Signal Cryptography. https://www.researchgate.net/profile/Aparna_Ramdoss/publication/323127358_Role_of_Windowing_Techniques_in_Speech_Signal_Processing_For_Enhanced_Signal_Cryptography/links/5a81c158a6fdcc6f3ead632d/Role-of-Windowing-Techniques-in-Speech-Signal-Processing-For-Enhanced-Signal-Cryptography.pdf.

[30] Esch T., Vary P. Efficient musical noise suppression for speech enhancement system. Proceedings of the 2009 IEEE International Conference on Acoustics, Speech and Signal Processing.

[31] Singhal S. High quality audio coding using multipulse LPC. Proceedings of the International Conference on Acoustics, Speech, and Signal Processing.

[32] Bradbury J. (2000). Linear Predictive Coding.

[33] O’Shaughnessy D. (1988). Linear predictive coding. IEEE Potentials.

[34] Chougala M., Kuntoji S. Novel text independent speaker recognition using LPC based formants. Proceedings of the 2016 International Conference on Electrical, Electronics, and Optimization Techniques (ICEEOT).

[35] Kim C., Seo K.D., Sung W. (2006). A robust formant extraction algorithm combining spectral peak picking and root polishing. Eurasip J. Appl. Signal Process..

[36] Snell R.C., Milinazzo F. (1993). Formant location from LPC analysis data. IEEE Trans. Speech Audio Process..

[37] Wald A. (1947). A note on Regression Analysis. Ann. Math. Stat..

[38] Lewis-Beck C., Lewis-Beck M. (2015). Applied Regression: An Introduction.

[39] Goldberger A.S. (1964). Econometric Theory.

[40] Seely J.F., El-Bassiouni Y. (1983). Applying Wald’s variance component test. Ann. Stat..

[41] Loy A., Follett L., Hofmann H. (2016). Variations of Q–Q Plots: The power of our eyes!. Am. Stat..

[42] Marden J.I. (2004). Positions and QQ plots. Stat. Sci..

[43] Poddar A., Sahidullah M., Saha G. (2019). Quality measures for speaker verification with short utterances. Digit. Signal Process..

[44] Dresch A.A.G. (2015). Método para Reconhecimento de Vogais e Extração de Parâmetros Acústicos para Analises Forenses. Master’s Thesis.

[45] Panayotov V., Chen G., Povey D., Khudanpur S., Vassil P. Librispeech: An ASR corpus based on public domain audio books. Proceedings of the 2015 IEEE International Conference on Acoustics, Speech and Signal Processing (ICASSP).

[46] Teyssier P. (1982). História da língua portuguesa, Lisboa.

[47] Schütz R. História da Língua Inglesa. https://www.sk.com.br/sk-historia-da-lingua-inglesa.html.

